# Fuel, cost, energy efficiency and CO_2_ emission performance of PCM integrated wood fiber composite phase change material at different climates

**DOI:** 10.1038/s41598-023-34616-8

**Published:** 2023-05-12

**Authors:** Nour Bassim Frahat, Abid Ustaoglu, Osman Gencel, Ahmet Sarı, Gökhan Hekimoğlu, Ali Yaras, Juan José del Coz Díaz

**Affiliations:** 1 Department of Civil and Architectural Constructions, Faculty of Technology and Education, Suez University, Suez, Egypt; 2 Department of Mechanical Engineering, Bartin University, 74100 Bartin, Türkiye; 3 Department of Civil Engineering, Bartin University, 74100 Bartin, Türkiye; 4 Department of Metallurgy and Materials Engineering, Karadeniz Technical University, Trabzon, Türkiye; 5 Department of Metallurgy and Materials Engineering, Bartin University, Bartin, Türkiye; 6 Department of Construction and Manufacturing, University of Oviedo, 33204 Gijón, Spain

**Keywords:** Energy science and technology, Engineering, Materials science

## Abstract

Wood fiber is a great potential supportive material for creating a new composite the phase change materials (PCM) due to its beneficial qualities, including high sorption competency, low density, enviro -friendliness, economic effectiveness, and chemical inertness. The main objective of this paper is to study the effect of using the wood fiber/eutectic mixture of stearic and capric acid on the fuel, cost, and carbon emission-saving potentials for various PCM cases. Which experiences a phase transition within the thermally pleasant temperature range of buildings, used for the building's thermal energy storing purposes and consumption cost saving. The energy performance analysis was carried out for buildings incorporated with stearic and capric acid eutectic mixture of PCM with wood fiber-based insulation material (INS) in different climate regions. The results showed that the largest energy-saving capacity belongs to PCM5. The energy saving reaches 52.7% for PCM5 for a thickness of 0.1 m. The PCM1, PCM2, PCM3, PCM4 can provide energy saving rates of 23.5%, 34.3%, 44.7% and 50.5%, respectively. INS-PCM5 can provide about 1.74-, 1.5-, and 1.33 times larger cost savings than INS in 2nd, 3rd, and 4th regions for all fuels. The payback period varies between 0.37 and 5.81 years regarding the fuel and Region. Finally, the results indicate that the proposed composite provided a promising energy-saving potential in building applications by reducing.

## Introduction

Technological developments in the last decade have significantly increased energy demand. It has environmental problems, global warming, and an energy crisis. Renewable energy sources and energy efficiency have become crucial issues because of the limited amount of fossil fuels. The structures are one of the largest energy consumers, accounting for about 40% of the global energy consumption. Developing and developed countries exert great efforts to generalize energy-efficient buildings and use renewable energy sources more effectively. It is a growing interest to transform structures into the net-zero energy building concept. These will contribute to a reduction in energy consumption and CO_2_ emissions.

It is a legal requirement to envelop the building with insulation materials. Therefore, most of the investments and expenses in the construction sector are allocated to the building envelope. This operation also provides energy control of the building as it adjusts the thermal energy requirements of indoor and outdoor spaces^1^. However, it does not present an energy-efficient building. On the other hand, renewable energies, especially solar energy, play an important role with a high privilege in energy-efficient construction. Efforts have been exerted to find innovative solutions and produce functional building materials using solar radiation.

One of the famous and practical techniques to enhance the energy performance of the building is phase change materials (PCMs) usage. PCMs change phase depending on temperature (from liquid to solid and contrarywise). Moreover, they have a high specific heat capacity and receive or give significant amounts of thermal energy during phase transition^2^. In other words, PMCs can store or release through the solidifying (discharging) and melting (charging) processes, respectively. By this means, the energy demand can be controlled, and the energy can be used more efficiently. With PMC applications in buildings, it is possible to shift the peak load to less-peak times for energy efficiency^3–5^. Therefore, many researchers focused on producing PCMs and their characterization from various raw materials and mixtures. Some of the produced PCMs and their latent heat values (J/g) are as follows; kaolin-based composites containing capric acid, PEG600, and heptadecane with latent heat around 27.23–34.63 kJ/kg^6^, eutectic composites (form-stabilized sepiolite and fatty acid) with 76.16 kJ/kg in cement based-plaster for indoor temperature regulation^7^, organic-modified montmorillonite composites with latent heat of 79.25 kJ/kg^8^, expanded perlite composite/ myristyl alcohol-capric acid having 93.9 kJ/kg phase change enthalpy for construction application^9^, stearic-lauric acid/expanded perlite composite with about 131 kJ/kg enthalpy^10^, diatomite/fatty acidform-stable composite with 87.33 kJ/kg^11^, composite containing paraffin and diatomite with 70.51 kJ/kg^12^, expanded vermiculite/paraffin composite with 137.6 kJ/kg^13^, polyethylene glycol (PEG600) with natural clay and gypsum with 28.79 kJ/kg^14^, dodecanol incorporated cement with 18.39 kJ/kg^15^ and n-octadecane-expanded graphene composite with 181.2 kJ/kg^16^. Considering the energy storage performances of the materials mentioned above, it is clear that they serve to decrease energy consumption. The melting temperature of PCM, PCM type, PCM quantity/thickness and position, and the subjected inside and outside environments affect PCM performance^17–19^. The construction and building materials such as hollow bricks^20,21^, concrete blocks^21,22^, pipes^23^, and wallboards^24^ containing PCMs were investigated in the literature. In recent years, the energy performances of buildings incorporating PCM into conventional building materials have been defined using various simulation and modeling tools^25–28^.

Sovetova et al.^29^ reported that up to 34% reduction in energy consumption occurs in buildings with PCM in eight hot cities. They also put forward that the increased surface area and decreased thickness of PCM would further enhance the building's energy efficiency. Alam et al.^30^ made a simulation analysis of eight cities in Australia. Using PMC in buildings can save up to 23% of energy. Ascione et al.^31^ examined the cooling load of the inner wall covered with FDM, which has 175 kJ/kg latent energy and 26–29 ℃ melting temperature for different climatic conditions. The results indicated that the demand for energy declines with increasing PCM thickness, and PCM does not exhibit the same efficiency for different climatic zones. Li and Chen^32^ conducted a numerical investigation on the Trombe wall, including PCM-encapsulated heat storage.

A wall including granular capsules with PCM was compared to that without PCM. PCM can provide about a 20% increment in the average indoor temperature at night. A thermal efficiency of 76% was achieved for the PCM heat storage wall. Pasupathy et al.^33^ evaluated the effect of the roof covered with a PCM on the thermal performance of the structure. Theoretical and experimental analyses were carried out to decide the optimum design. The ceiling temperature stayed steady at 27 ℃ during the day for the PCM-using room, while it significantly fluctuated in non-PCM rooms. The environmental conditions slightly affect the interior temperature due to the energy absorption of PCM. Xie et al.^34^ made a thermal performance analysis of PCM to lessen the structure's energy consumption. Five PCM wallboards were considered on the exterior wall, and their thermal performance on building energy performance was analyzed for a year. They found that the PCM wallboard significantly affected the commission in June and September. The building energy consumption with PCM was about 103 kJ lower than the reference wallboard. Izquierdo-Barrientos et al.^35^ analyzed the exterior surface of a structure wall containing PCM by changing the PCM layer position, wall orientation, environments, and phase change temperature.

The PCM integration diminished sudden heat flux variation throughout the wall for the appropriate PCM melting temperature selection regarding the season and wall orientation. Dong et al.^36^ conducted a numerical analysis of the thermal performance of PCM-containing roofs for the cold Region of China. The effect of solar radiation, latent heat, PCM layer thickness, and roof surface absorptivity were considered as calculation parameters. The results indicate that PCM was delayed about 3 h to reach the peak temperature. Salihi et al.^37^ performed a numerical study on a building with PCM-integrated walls in semi-arid climates. A parametric performance evaluation of temperature, thickness, location, wall configuration, and mechanical ventilation effect changes was performed. The results specified that the proposed component with PCM could reduce the cooling-heating loads. RT-28 was selected as the optimal PCM for the semi-arid Region with the most considerable average temperature fluctuation reduction of 1.91 ℃.

The use of double and triple-layer PCM provided about 7.3% and 15.2% reduction in energy consumption. Heim and Clarke^38^ performed an energy analysis of PCM-gypsum with ESP-r. PCM-gypsum composite was considered on the internal room lining. The result indicated that PCM-gypsum panels could lessen thermal energy load by up to 90% in cold seasons. Biswas et al.^39^ performed numerical and experimental analyses of nano-PCM added wallboard. A nano-PCM with a highly conductive expanded graphite nanosheet was founded. The experiment was carried out in an outdoor and hot-humid region. According to the annual evaluation, nano-PCM wallboard reduced energy consumption for air-conditioning. Depending on wall orientation, the heating and cooling load is reduced by 10–20%.

Due to their beneficial qualities, including elevated absorption competency, lower density, enviro-friendliness, economic efficiency, and chemical inertness, wood fiber and flour are widely utilized in many construction applications^40^. It contains lignocellulose fibers with lots of hydroxyl groups^41,42^. This chemical composition makes it an excellent supporting component to improve composite phase change materials (CPCMs) having leak-proof properties. These kinds of composites are utilized to give wooden elements thermal functionality.

Ma et al.^43^ proposed lauric acid-myristic acid/wood flour mixtures having proper phase change features.

They stated that the proposed composites showed excellent thermal consistency after 500 cycles and fine practical potential for thermal energy storage. Liang et al.^44^ prepared a form stable CPCM including wood flour/fatty acid with direct impregnation. Wood flour was used to be a supporting material. Fatty acid/wood flour CPCMs revealed complete thermal consistency and steadiness free from leakage. Ma et al.^45^ used capric acid-palmitic acid with delignified wood to attain form-stable CPCM having appropriate TES properties. Delignified-wood has a porous honeycomb structure and provides better adsorption characteristics than wood. Jeong et al.^46^ formed thermally improved wood flooring through microencapsulated PCM. It is observed that MWAC with MPCM could be used for wood-based flooring due to its muscular bonding strength and good energy storage capacity. Barreneche et al.^47^ combined organic PCM and wood. To eliminate the leakage problem of PCM, a polystyrene solution was applied to the final product. The proposed composite provided a promising energy-saving potential in a building application. Mathis et al.^48^ prepared wood boards, including biobased PCM. It is observed that wood boards provide high energy-saving potential in buildings due to significant thermal energy-storing capacity with about 22 ℃ phase change temperature.

In conclusion, many papers deal with several experimental studies and analyses about synthesizing novel PMCs, their characterization, and their incorporation into building materials. The outputs of these papers also revealed advantageous influences of the utilization of PMCs, such as improving thermal comfort and heating/cooling energy savings.

Using wood fiber (WF) as an enviro- and eco-friendly support component for investigating a new composite PCM can significantly influence TES applications in wooden structures. Therefore, a wood fiber was combined with a eutectic combination of stearic-capric acid, which experiences a phase transition within the thermally pleasant temperature range of buildings. In this study, a novel composite PCM with wood fiber proposed was considered an energy storage component of a building. Moreover, studies have yet to focus on the effect of construction and building materials containing phase change materials on fuel types and consumption, cost saving, and emission values by considering different climates.

## Methodology

### PCM material

The thermal achievement of a passive structure design may be improved by using PCM as thermal energy storage. PCM-impregnated insulation material was considered for evaluation. The stearic-capric acid (SA-CA) eutectic mixture impregnated Wood fiber (WF) insulation material was supposed to improve the energy performance of a building.

The melting temperature was around 29–32 ℃ in the case of capric acid. That of stearic acid was around 69–72 ℃. The purities of capric and stearic acids were higher than 98% and 97%, respectively. The ratios of CA and SA in the eutectic mixture were 83 and 17 wt.%. The eutectic mixture melts at the temperature of 24.73 ℃. The eutectic mixture's latent heat is 179.10 J/g. The mixture's solid and liquid state densities were 983 kg/m^3^ and 853 kg/m^3^. The insulation material with PCM (INS-PCM) was produced by impregnating the SA-CA mixture into wood fiber (WF) for various PCM. The WF/SA-CA mixture at the selected composition ratio was stirred in acetone for 2 h for homogenization. These eliminate the inside air of the beaker and accomplish better impregnation. The attained sol–gel was maintained in a temperature-managed vacuum oven at 30 °C for 30 min. After that, the WF/SA-CA composite was subjected to heat treatment at 80 °C for 3 h to evaporate its acetone content. After the molding process of the sample with a size of 0.02 m × 0.025 m × 0.005 m, it was then subjected to a leakage test by heated at 30 °C to decide the most significant impregnation ratio of PCM ingredient. The impregnation-molding and leakage test procedure was repeated, and for different PCM, weight contents changed from 25 to 52 wt.%. The PCM's incorporation ratio into the composite was obtained with the following:

1 $$ Incorporation\,PCM\,(wt.\% ) = \frac{{m_{PCM} }}{{m_{PCM} + m_{WF} }} \times 100 $$

where *m*_PCM_ and *m*_WF_ are designated as mass quantities of the WF and PCM ingredients of the composite. m_PCM_ was decided by considering the weight loss in the sample composite after the leakage test, and this value was used to calculate the PCM's incorporation ratio via this equation. The thermal conductivity of stearic-capric acid (SA-CA) eutectic mixture/wood fiber (WF) can be obtained by:

2 $$ k_{WF\& PCM} = k_{WF} R_{wt\% WF} + k_{PCM} R_{wt\% PCM} $$

**Figure 1:**
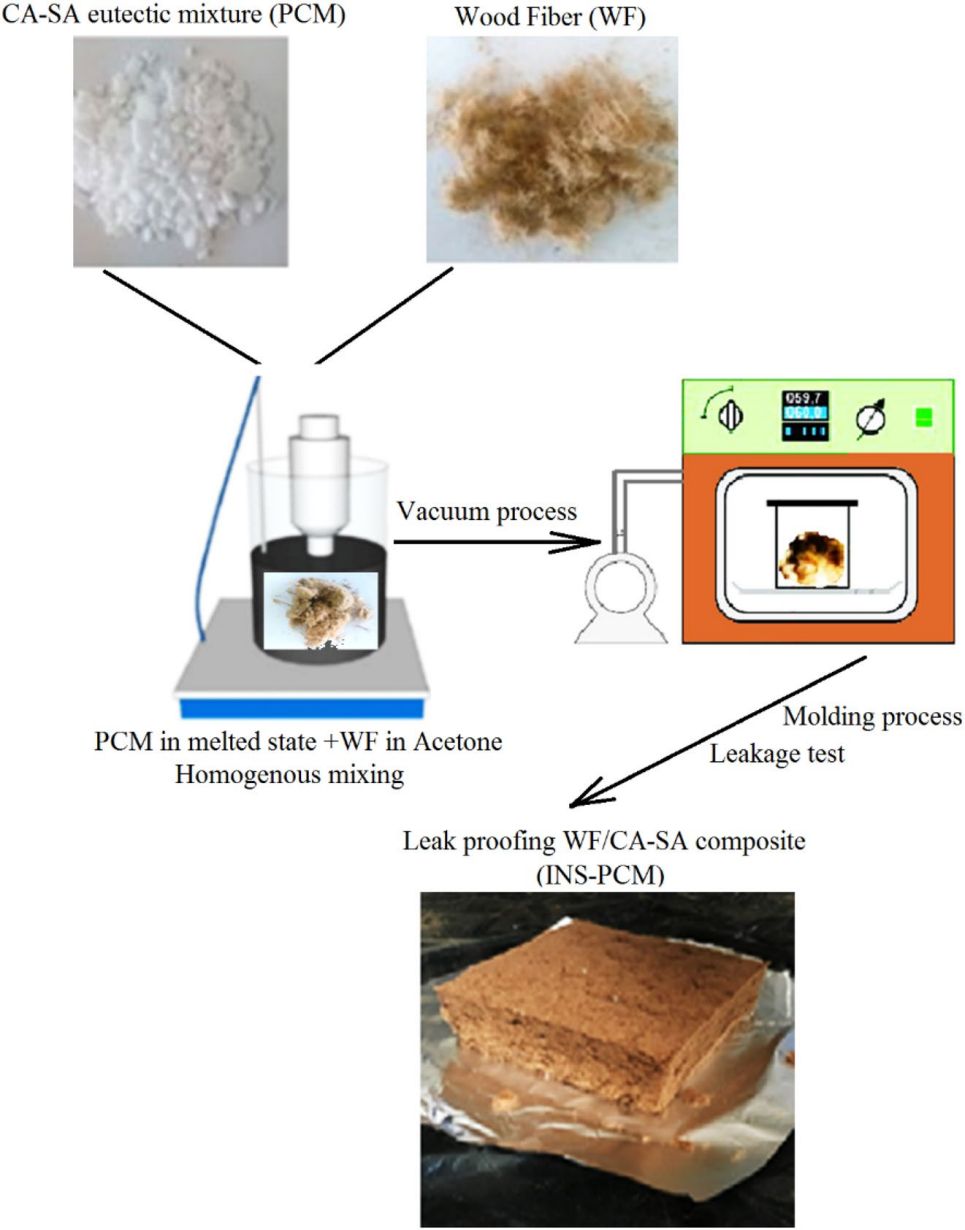
Photograph image of the final product (INS-PCM) and the procedure schema used to prepare leak-proofing WF/CA-SA composite.

The weight loss of 25 wt.%, 35 wt.%, 45 wt.%, 50 wt.%, and 52 wt.% PCM cases were decided to be less than 0.1% in the leakage test. Consequently, the composite sample, which contains 52wt% PCM and has a weight loss of less than 1%, was identified as a leakage-proofing sample and is called INS-PCM in this work (Fig. 1). After the wood fiber 52, wt.% PCM ratio, the amount of PCM leakage significantly increased. Therefore, 25, 35, 45, 50, and 52 wt.% were selected for the investigation.

The energy storage specification of the PCM and PCM-impregnated wood fiber-based insulation components were defined by the Differential Scanning Calorimetry (DSC) (Table 1). Figure 1 shows the final product (INS-PCM) and the procedure schema for preparing leak-proofing WF/SA-CA.

**Table 1:** Thermophysical properties of thermal energy storage capacity of wood fiber/ eutectic mixture of stearic-capric acids^49^.

Label	CPCM	Thermal conductivity k (W/mK)	Freezing temperature (℃)	Melting temperature (℃)	Melting enthalpy (kJ/kg)
PCM	CA-SA eutectic mixture	0.14	23.7	24.7	179.1
INS + PCM1	WF/(CA-SA)(25 wt%)	0.0635	20.2	24.2	44.5
INS + PCM2	WF/(CA-SA)(35 wt%)	0.0737	21.8	23.0	61.7
INS + PCM3	WF/(CA-SA)(45 wt%)	0.0839	22.1	24.0	79.9
INS + PCM4	WF/(CA-SA)(50 wt%)	0.0890	22.6	23.3	88.8
INS + PCM5	WF/(CA-SA)(52 wt%)	0.0910	21.9	23.4	92.1

### Solar energy analysis

The PCM-impregnated wood fiber-based insulation material was considered on the structure's wall, ceiling, and floor. The PCM receives solar radiation throughout the year. Therefore, it is essential to decide on incident energy. Solar energy illuminates the structure's walls and ceiling. The following equation shows monthly mean daily (MMD) irradiation on an inclined plane^50–52^.

3 $$ \overline{q}_{s} = \overline{H}_{b} \overline{R}_{b} + \overline{H}_{d} R_{d} + \overline{H}\rho_{r} R_{r} $$

where the *H*_b_, *H*_d_, and *H* are direct, diffuse, and global irradiation on the horizontal plane. The upper line on the symbols indicates the MMD values. Global solar radiation is measured using a pyranometer. *R*_b_, *R*_d_, and *R*_r_ are the angle factors of direct, diffuse, and reflected radiation. The angle factor in the solar radiation equation can be rewritten as^50,51^.

4 $$ \overline{q}_{s} = \overline{H}_{b} \cos \overline{\theta }\sin \overline{\alpha }_{S}^{ - 1} + \overline{H}_{d} \cos^{2} \left( {0.5\beta } \right) + \overline{H}\rho_{r} \sin^{2} \left( {0.5\beta } \right) $$

Diffuse and reflected solar radiation is associated with the tilt angle of a plane. The building surface can be considered perpendicular. The angle factor of direct radiation is a function of latitude*ϕ*, tilt angle*β*, declination*δ*, and sunset hour angle *ω*_S_^50^.

5 $$ \overline{R}_{b} = \frac{{\left( {{{\omega^{\prime}_{S} \pi } \mathord{\left/ {\vphantom {{\omega^{\prime}_{S} \pi } {180}}} \right. \kern-0pt} {180}}} \right)\sin \left( {\phi - \beta } \right)\sin \delta + \cos \left( {\phi - \beta } \right)\cos \delta \sin \omega^{\prime}_{S} }}{{\left( {{{\omega_{S} \pi } \mathord{\left/ {\vphantom {{\omega_{S} \pi } {180}}} \right. \kern-0pt} {180}}} \right)\sin \phi \sin \delta + \cos \phi \cos \delta \sin \omega_{S} }} = \frac{{\cos \overline{\theta }}}{{\sin \overline{\alpha }_{S} }} $$

where, *ω*_S_ and *ω'*_S_ are the sunset hour and sunset hour angles for the titled plane for the mean mount day, respectively. These can be calculated by^50^

6 $$ \omega^{\prime}_{S} = \min \left[ {\begin{array}{*{20}c} {\cos^{ - 1} \left( { - \tan \phi \tan \delta } \right)} \\ {\cos^{ - 1} \left( { - \tan \left( {\phi - \beta } \right)\tan \delta } \right)} \\ \end{array} } \right] $$

7 $$ \omega_{S} = \cos^{ - 1} \left( { - \tan \phi \tan \delta } \right) $$

The MMD direct irradiation on the horizontal plane is calculated with

8 $$ \overline{H}_{b} = \overline{H} - \overline{H}_{d} $$

where MMD diffuse radiation is calculated by;

9 $$ \overline{H}_{d} = \overline{H}\left( {1 - 1.097\overline{K}_{T} } \right) $$

The clearness index is designated by the rate of MMD global irradiation on the horizontal plane to that of extraterrestrial irradiation.

10 $$ \overline{K}_{T} = {{\overline{H}} \mathord{\left/ {\vphantom {{\overline{H}} {\overline{H}_{0} }}} \right. \kern-0pt} {\overline{H}_{0} }} $$

where the MMD extraterrestrial radiation is obtained by;

11 $$ \overline{H}_{0} = \frac{24}{\pi }I_{SC} \left( {1 + 0.034\cos \left( {\frac{360n}{{365}}} \right)} \right)\left( {\sin \delta \sin \phi \frac{\pi }{180}\omega_{SS} + \cos \delta \cos \phi \sin \omega_{SS} } \right) $$

where* I*_SC_ is the solar constant. *n* is the number of days in a year. Table 2 shows the typical days of a month, declination angle, sunset angle, sunset angle for the titled surface, and angle factor of direct radiation.

**Table 2:** Typical days in the months, declination, sunset angles, and *R*_b_ values.

Mon	*n*	*δ*	*W*_ss_ 1st region	*W*_ss_ 2nd region	*W*_ss_ 3rd region	*W*_ss_ 4th region	*w*_ss'_1st region	*W*_ss'_ 2nd region	*W*_ss'_ 3rd region	*W*_ss'_ 4th region	*R*_b_ 1st region	*R*_b_ 2nd region	*R*_b_ 3rd region	*R*_b_ 4th region
1	17	− 20.92	73.3	70.6	71.3	71.4	120.6	116.1	117.1	117.2	2.04	2.42	2.31	2.31
2	47	− 12.95	80.1	78.5	78.9	78.9	107.8	105.4	105.9	106.0	1.43	1.65	1.59	1.59
3	75	− 2.42	88.2	87.9	88.0	88.0	93.2	92.8	92.9	92.9	0.86	0.99	0.96	0.95
4	105	9.41	97.1	98.3	98.0	98.0	77.2	79.0	78.6	78.6	0.39	0.47	0.45	0.44
5	135	18.79	104.8	107.2	106.6	106.5	63.0	66.9	66.0	66.0	0.07	0.13	0.11	0.11
6	162	23.09	108.7	111.7	110.9	110.9	55.4	60.6	59.4	59.4	− 0.06	− 0.01	− 0.03	− 0.03
7	198	21.18	106.9	109.7	109.0	108.9	58.9	63.5	62.5	62.4	0.00	0.05	0.04	0.03
8	228	13.45	100.3	102.0	101.6	101.5	71.4	74.0	73.4	73.4	0.25	0.31	0.30	0.30
9	258	2.22	91.7	91.9	91.9	91.9	87.0	87.4	87.4	87.3	0.66	0.77	0.74	0.74
10	288	− 9.60	82.7	81.6	81.9	81.9	103.0	101.2	101.6	101.7	1.22	1.41	1.36	1.36
11	318	− 18.91	75.1	72.7	73.3	73.3	117.2	113.2	114.1	114.2	1.87	2.19	2.10	2.10
12	344	− 23.05	71.4	68.3	69.1	69.2	124.5	119.3	120.5	120.6	2.26	2.69	2.57	2.56

**Figure 2:**
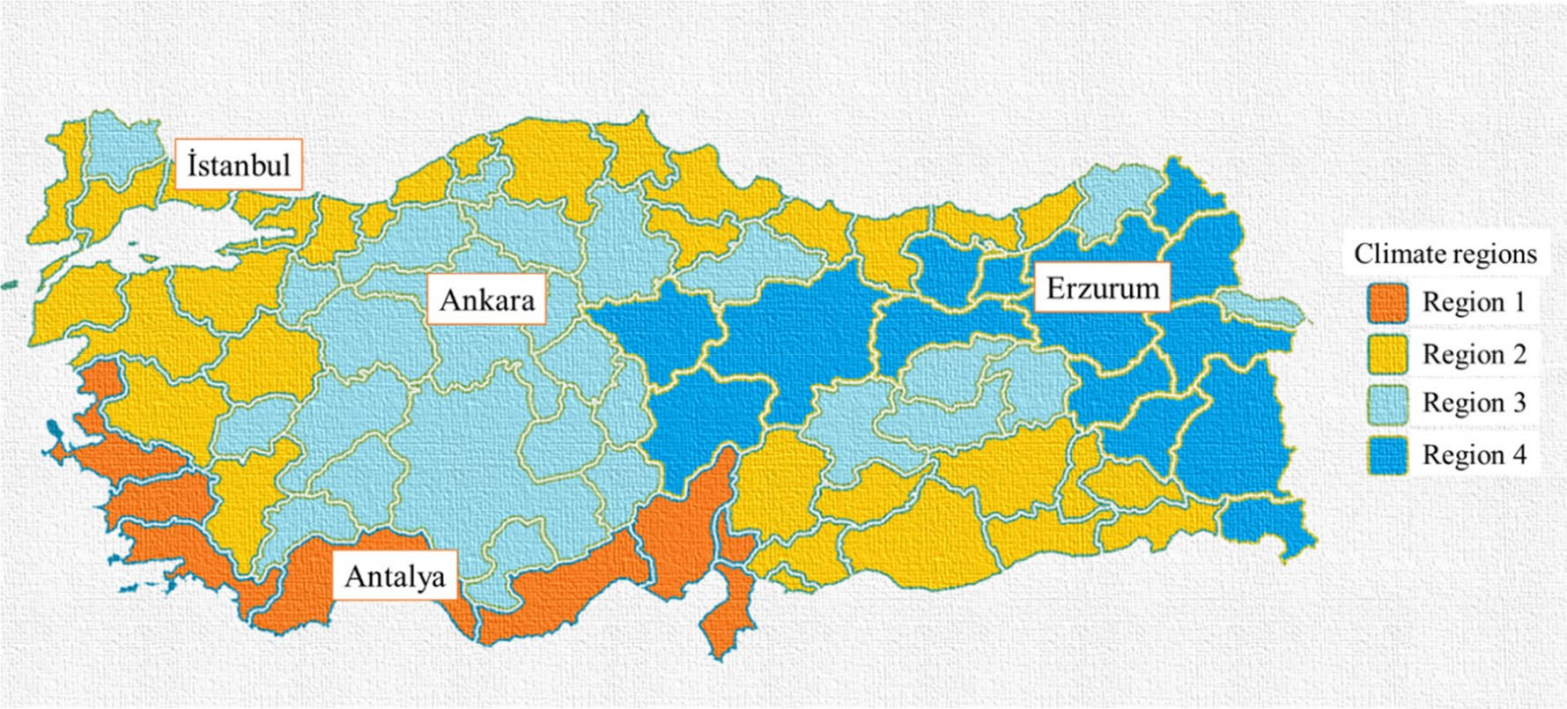
Climate regions of Türkiye (TS 825)^53^.

Turkey’s thermal insulation standards for buildings (TS 825) divide the country into four climate areas based on the climate environments^53^. Four climate regions in Turkey were considered for evaluating a building’s energy efficiency. The climate is typically milder along the coast. Mediterranean climate is prevalent throughout the shores of the Mediterranean and Aegean Seas. As a result, the midsummers are warm and arid, and the winters are mildly cold and damp. The Black Sea’s coastal region experiences an oceanic climate with hot, rainy summers and cold, rainy winters—the plateau in central Anatolia experiences continental weather. Winters are chilly and covered in snow, and summers are warm and arid. Eastern Anatolia experiences harsh winters that are exceedingly cold and snowy, as well as sweltering and dry summers. The four cities chosen are Ankara, Istanbul, Erzurum, and Antalya. These are in the central Anatolian plateau, in the coastal region bordering the Black Sea, in eastern Anatolia, and the coastal region bordering the Mediterranean Sea, respectively. These cities are in the 1st, 2nd, 3rd, and 4th climate regions. Figure 2 exhibitions the cities' positions about four climate regions regarding Turkey's thermal insulation standards for buildings (TS 825)^53^.

### Thermal analysis

A building component may include convective, conductive, and radiative heat transfer mechanisms. Heat transfer occurs over walls, windows, doors, ceilings, floors, and ventilation. The heat transfer rate for the planar surface is described in the following:

12 $$ \dot{Q} = UA_{s} \Delta T = \frac{\Delta T}{R} = \frac{{T_{in} - T_{out} }}{{\frac{1}{{h_{in} A_{s} }} + \sum\limits_{i = 1}^{i = n} {\frac{{d_{i} }}{{k_{i} A_{s} }}} + \frac{1}{{h_{out} A_{s} }}}} $$

When calculating the thermal requirement of a structure, the thermal acquisition, the thermal acquisition, containing inner ones associated with solar radiation (alternating with building orientation, location, and window area), electrical devices, and household appliances should be considered, as well as the heat loss over the components of the structure. The yearly thermal necessity of a building can be achieved by considering the energy losses and gains in the system. The convective heat transfer can be attained by using the following;

13 $$ {\varvec{Nu}} = \frac{{h_{m} L_{c} }}{{k_{m} }} $$

The thermal conductivity of a component, *k*_m,_ varies according to the conditions. The characteristic length, *L*_c,_ describes the scale of a physical system. The total specific heat loss is described by^53–55^

14 $$ H = H_{T} + H_{V} = \sum {U_{{\user2{comp,i}}} A_{{\user2{comp,i}}} } + \rho cV^{\prime} $$

*H*_T_ and *H*_V_ indicate heat losses through building components and ventilation, respectively. The ventilation loss is determined using heat capacity c, airflow rate *V*ʹ, and density*ρ*. *U*_comp, i_ and *A*_comp,i_ are the total heat transfer coefficient, and the area of *i* is the component. The building thermal insulation requirements standard indicates that the internal heat gains can be considered 5 W/m^2^ at the utmost in a typical building^53^. The internal heat gains can be obtained by totaling inner and solar thermal gains as follows;^53,56^

15 $$ \phi_{T,m} = \phi_{s,m} + \phi_{i,m} = \sum {r_{i,m} g_{i,m} I_{i,m} A_{w} } + 5A_{n} $$

These can be decided by considering the shading factor of window *r*_*i,m*_, the transmissivity through the window *g*_*i,m*_, and the incident irradiation *I*_i,m_ for the i-direction. *A*_*w*_, and *A*_*n*_ is the window and building areas in use. The thermal energy necessity of a structure can be determined annually by;^53,57^

16 $$ \dot{Q}_{an} = \sum\limits_{i = 1}^{i = 12} {\dot{Q}_{i,m} } = \sum\limits_{i = 1}^{i = 12} {\left[ {H(T_{in,m} - T_{out,m} ) - (\phi_{T,m} )\left( {1 - \exp \left( {\frac{{H(T_{out,m} - T_{in,m} )}}{{\phi_{T,m} }}} \right)} \right)} \right]t} $$

PCM absorbs energy regarding its latent heat and releases the stored energy at a lower than its freezing temperature. The inside temperatures of buildings were considered above the melting temperature in the daytime while reducing to below the freezing temperature at nighttime. The PCM-impregnated wood fiber-based insulation material was supposed to cover the building’s inside walls, floor, and ceiling for maximum storage energy utilization. The energy storage in the liquid phase of PCM was ignored. Thus, the buildings can store the PCM latent heat for a day at most. PCM energy storage capacity was considered the energy gain to achieve the annual thermal requirement of the structure. The yearly energy needs of PCM-impregnated insulation-integrated buildings can be decided by;

17 $$ \dot{Q}_{an} = \sum\limits_{i = 1}^{i = 12} {\dot{Q}_{i,m} } = \sum\limits_{m = 1}^{m = 12} {\left[ {H(T_{in,m} - T_{out,m} ) - (\phi_{T,m} )\left( {1 - \exp \left( {\frac{{H(T_{out,m} - T_{in,m} )}}{{\phi_{T,m} }}} \right)} \right)} \right]t} - h_{fg} m_{PCM} n_{d,m} $$

where *h*_fg_, *m*_PCM_, and *n*_d_ are the phase change enthalpy, the amount of PCM in the structure, and the number of days in a month, respectively. The yearly fuel price of a structure is obtained by considering the fuel type, the fuel's net calorific value (NCV), fuel efficiency, and the total heat needed^58^. The energy cost of the structure heating requirement is calculated by the following;

18 $$ C = \frac{{\dot{Q}_{an} }}{NCV}c $$

where *c* is the unit cost of the fuel. Natural gas, LPG, coal, fuel oil, and electricity were selected. The annual fuel amount is achieved by the yearly energy requirement rate to the fuel's net calorific value. The calorific values, efficiencies, and fuel unit cost are shown in Table 3^56,58^. The Nusselt numbers related to the building components are specified in Table 4. Table 5 shows the monthly average outdoor temperatures of four climate regions. Table 6 states building component surface areas, component thicknesses, conductivities, and some properties of building components.

**Table 3:** Fuel efficiency, calorific values, and unit price of fuels^56,58^.

Fuel efficiency		Calorific values			Price		
*η*_coal_	0.65	*Hu*_coal_	6.98	kWh/kg	*C*_coal_	152	$/tonne
*η*_elec_	0.99	*Hu*_elec_	1	kWh/kWh	*C*_elec_	0.074900	$/kWh
*η*_foil_	0.82	*Hu*_foil_	10.69	kWh/kg	*C*_foil_	0.622	$/kg
*η*_lpg_	0.92	*Hu*_lpg_	12.76	kWh/kg	*C*_lpg_	1.25	$/m3
*η*_ng_	0.92	*Hu*_ng_	9.59	kWh/Nm3	*C*_ng_	0.14720	$/m3
		*Hu*_ng_	13.1	kWh/kg

**Table 4:** Heat transfer coefficients for building components.

	Nusselt number	Conditions and calculations
Wall outdoor for (vertical plane)	Combined laminar and turbulent $(0.037{\text{Re}}_{L}^{0.8} - 871) \times \Pr^{1/3}$	0.6 ≤ Pr ≤ 60 5 × 10^5^ ≤ Re_L_ ≤ 10^7^ ${\text{Re}}_{L} = {{V_{{{\text{air}}}} L_{c} } \mathord{\left/ {\vphantom {{V_{{{\text{air}}}} L_{c} } \nu }} \right. \kern-0pt} \nu }$
Wall indoor (vertical plate)	$\left\{ {0.825 + \frac{{0.0378{\varvec{Ra}}^{1/6} }}{{\left[ {1 + \left( {0.492/\Pr } \right)^{9/16} } \right]^{8/27} }}} \right\}^{2}$	Rayleigh number $Ra = \frac{{g\beta (T_{room} - T_{wall} )L_{c}^{3} }}{{\nu^{2} }}\Pr$
An air gap of double window	$0.42Ra_{L}^{1/4} \Pr^{0.012} (\frac{H}{L})^{ - 0.3}$	10 ≤ $\frac{H}{L}$ ≤ 40 1 ≤ Re_L_ ≤ 2 × 10^4^ 10^4^ ≤ Ra_L_ ≤ 10^7^ $\dot{Q}_{{{\varvec{wi}}}} = n_{{{\varvec{wi}}}} A_{wi} \left( {kNu\frac{\Delta T}{{L_{c} }} + \frac{{\sigma \left( {T_{s1} - T_{s2} } \right)}}{{{1 \mathord{\left/ {\vphantom {1 {\varepsilon_{s1} }}} \right. \kern-0pt} {\varepsilon_{s1} }} + {1 \mathord{\left/ {\vphantom {1 {\varepsilon_{s2} - 1}}} \right. \kern-0pt} {\varepsilon_{s2} - 1}}}}} \right)$
Roof and ceiling (Lower hot surface)	$0.27\left( {g\beta (T_{room} - T_{ceiling} )Lt^{3} \nu^{ - 2} \Pr } \right)^{1/4}$	10^5^ < Gr Pr < 10^11^ $U = \sum {f_{a,i} U_{i} = } (U_{f,a} )_{as} R_{i} + (U_{f,a} )_{stud} R_{i}$ $\varepsilon_{effective} = \left( {1/\varepsilon_{1} + 1/\varepsilon_{2} - 1} \right)^{ - 1}$
Floor (Upper hot surfaces)	$0.15\,\left( {g\beta (T_{room} - T_{floor} )Ld^{3} \nu^{ - 2} \Pr } \right)^{1/3}$	10^7^ < Gr Pr < 10^11^
Door	$\left\{ {0.825 + \frac{{0.0378{\varvec{Ra}}^{1/6} }}{{\left[ {1 + \left( {0.492/\Pr } \right)^{9/16} } \right]^{8/27} }}} \right\}^{2}$	$Ra = \frac{{g\beta (T_{room} - T_{wall} )L_{c}^{3} }}{{\nu^{2} }}\Pr$

**Table 5:** Monthly average outdoor temperatures of four climate regions^53^.

	Months	1st region	2nd region	3rd region	4th region
1	*T*_ave, Jan_	8.4	2.9	− 0.3	− 5.4
2	*T*_ave, Feb_	9	4.4	0.1	− 4.7
3	*T*_ave, Mar_	11.6	7.3	4.1	0.3
4	*T*_ave, Apr_	15.8	12.8	10.1	7.9
5	*T*_ave, May_	21.2	18	14.4	12.8
6	*T*_ave, Jun_	26.3	22.5	18.5	17.3
7	*T*_ave, July_	28.7	24.9	21.7	21.4
8	*T*_ave, Aug_	27.6	24.3	21.2	21.1
9	*T*_ave, Sep_	23.5	19.9	17.2	16.5
10	*T*_ave, Oct_	18.5	14.1	11.6	10.3
11	*T*_ave, Nov_	13	8.5	5.6	3.1
12	*T*_ave, Dec_	9.3	3.8	1.3	− 2.8

**Table 6:** Areas *A*_i_, thicknesses *d*_i_, and properties of building components.

		Unit			Unit			Unit	Areas		Unit
Wall											
* d*_plaster in_	0.02	m	*k*_plaster in_	0.87	W/m℃	*T*_in_	19	℃	*A*_door_	3.45	m^2^
* d*_plaster out_	0.02	m	*k*_plaster out_	0.87	W/m℃	*T*_wall in_	15	℃	*A*_window_	60.66	m^2^
* d*_gasconcrete_	0.2	m	*k*_gasconc_	0.32	W/m℃	*T*_wall out_	12.5	℃	*A*_column_	36	m^2^
* d*_ins_	0.05	m	*k*_ins_	0.0635	W/m℃	*P*_air_	101.325	kPa	*A*_column2_	38.4	m^2^
* d*_pcm_	0.05	m	*k*_pcm_	0.14	W/m℃	*V*_wind_	8.33	m/s	*A*_column3_	74.4	m^2^
			*k*_air_	0.02467					*A*_wall_	442.1	m^2^
Window									*A*_floor_	131.2	m^2^
* d*_airw_	0.02	m	*k*_airp_	0.02416	W/m℃	*T*_win in_	15	℃	*A*_build_	580.6	m^2^
* L*_w_	1.4	m	*F*w	0.8		*T*_win out_	12.5	℃	*A*_ceiling_	131.2	m^2^
			*τ*	0.75					*A*_net_	503.8	m^2^
			*g*	0.5					*A*_win east_	23.94	m^2^
Door									*A*_win west_	0	m^2^
* d*_door_	0.05	m	*k*_door_	4	W/m℃				*A*_win north_	17.8	m^2^
*L*_door_	2.3	m	*k*_aird_	0.02488	W/m℃				*A*_win south_	18.36	m^2^
Roof and ceiling								Building size			
*d*_conc_	0.2	m	*k*_conc_	1.3	W/m℃	*f*1	0.82		a	15.98	m
*d*_ins_	0.05	m	*k*_ins_	0.0635	W/m℃	*f2*	0.18		b	8.21	m
			*U*_atstud_	0.4484	W/m^2^℃	*J*1	0.9		h	12	m
			*U*_btwstud_	0.5371	W/m^2^℃	*J*2	0.9				
Floor											
* d*_wood_	0.02	m	*k*_wood_	2	W/m℃	*L*_floor_	3.995	m			
* d*_alum_	0.2	m	*k*_alum_	2.1	W/m℃						
* d*_tesbet_	0.05	m	*k*_tesbet_	1.74	W/m℃						
* d*_ferrocon_	0.2	m	*k*_ferrocon_	2.1	W/m℃						
* d*_plasterfl_	0.005	m	*k*_plasterfl_	0.87	W/m℃						
* d*_ins_	0.05	m	*k*_ins_	0.0635	W/m℃						

### Calculations of carbon emissions

The quantities of CO_2_ and SO_2_ emissions vary depending on combustion situations and the quantity of carbon and sulfur in the fuels. The chemical reaction for the complete combustion of any fuel with air follows^59^;

19 $$ C_{x} H_{y} O_{z} S_{t} N_{w} + \alpha .A\left( {3.76N_{2} + O_{2} } \right) \to xCO_{2} + \frac{y}{2}\left( {H_{2} O} \right) + tSO_{2} + BO_{2} + DN_{2} $$

where *x*, *y*, *z*, *t*,* w* represents the components number of the fuels. *A*, *B*, and *D* are stoichiometric coefficients and are calculated according to the above reaction as follows;

20 $$ A = \left( {x + \frac{y}{4} + t - \frac{z}{2}} \right) $$

21 $$ B = \left( {\alpha - 1} \right)\left( {x + \frac{y}{4} + t - \frac{z}{2}} \right) $$

22 $$ D = 3.76 \cdot \alpha \left( {x + \frac{y}{4} + t - \frac{z}{2}} \right) + \frac{w}{2} $$

CO_2_ and SO_2_ emission amounts were calculated, and NO_2_ was neglected. Therefore, the emissions per 1 kg of fuel;

23 $$ M_{{CO_{2} }} = \frac{{x \cdot CO_{2} }}{M}\left( {\frac{{kgCO_{2} }}{kgfuel}} \right) $$

24 $$ M_{{SO_{2} }} = \frac{{t \cdot SO_{2} }}{M}\left( {\frac{{kgSO_{2} }}{kgfuel}} \right) $$

The emission amounts of CO_2_ and SO_2_ for the total fuel (*M*_*fuel*_), respectively.

25 $$ M_{{CO_{2} }} = \frac{x \cdot 44}{M} \cdot M_{fuel} \left( {\frac{kg}{{m^{2} \cdot year}}} \right) $$

26 $$ M_{{SO_{2} }} = \frac{t \cdot 64}{M} \cdot M_{fuel} \left( {\frac{kg}{{m^{2} \cdot year}}} \right) $$

The molecule weight of the fuel, *M* is attained for each fuel by;^59^.

27 $$ M = 12 \cdot x + y + 16 \cdot z + 32 \cdot t + 14 \cdot w\left( {\frac{kg}{{kmol}}} \right) $$

The amount of carbon saving was calculated regarding fuel reduction based on the structure's yearly energy storage capacity. Table 7 shows the chemical formulas of four types of fuels. The solar radiation, thermal analysis, and carbon emission calculation were written in Engineering Equation Solver (EES) program. Thermo-physical properties of air were obtained from the EES library. These were used to attain heat loss through building components.

**Table 7:** Chemical formulas of fuels^60,61^.

Fuel type	Chemical formula
Coal	C_5.85_H_5.26_O_1.13_S_0.008_N_0.077_
Fuel oil	C_7.3125_H_10.407_O_0.04_S_0.026_N_0.02_
Natural gas	C_1.05_H_4_O_0.034_N_0.022_
LPG	C_3.7_H_4.1_

## Results and discussion

### Solar energy amount on the building surface

The latitudes of the cities in different climate regions are 36.89° N (1st), 40.98° N (2st), 37.97° N (3st), and 39.91° N (4st Region). To decide the MMD values of the solar radiation, the typical day of each month was considered^50^. The buildings were deemed located in regions with mostly light-colored buildings.

**Figure 3:**
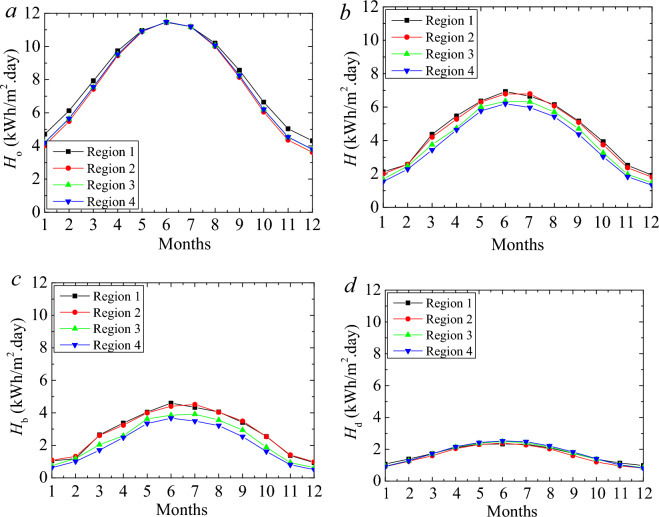
MMD global (**a**), extraterrestrial (**b**), direct (**c**), and diffuse (**d**) solar irradiation on the selected climate regions.

**Figure 4:**
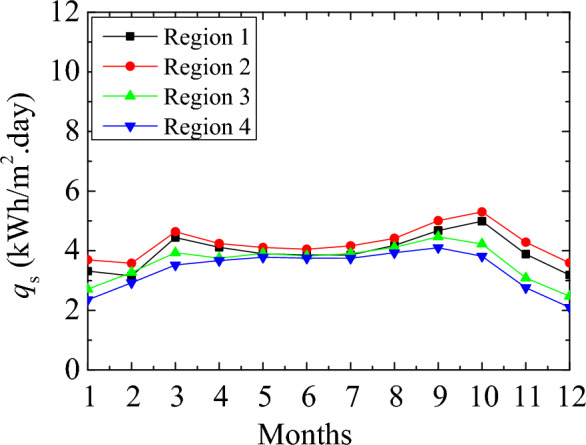
MMD solar irradiation on a perpendicular surface.

Figure 3 shows the MMD solar radiation in different climate regions. The MMD extraterrestrial and global irradiation on the horizontal plane is shown in Fig. 3a,b. Global solar irradiation includes direct and diffuse irradiations on the horizontal plane and is measured by a pyranometer. These values are available in the literature^62^. The extraterrestrial solar radiation values are similar in the summer months as the difference regarding the Region gets more considerable in the winter months. The global solar radiation in different regions has more clear differences. The maximum value is observed in June. Figure 3c,d illustrates the direct and diffuse irradiations. The magnitudes of the natural solar radiation in the 1st and 2nd regions are similar. The lowest value is observed in the 4th Region. By considering the direct, diffuse, and global solar radiation, the heat flux on a perpendicular surface of the building can be decided. Figure 4 indicates the various regions' MMD solar irradiation on the vertical surface. The magnitude of the solar heat flux in the summer months is observed as comparatively low since the sun rises higher in the sky, and the solar radiation strikes the ground at a higher angle compared to the winter months. However, the 1st Region has a more significant ambient temperature, the most extensive solar radiation in the 2nd Region. It is about the characteristic condition of the location. The annual average daily values were 3.96, 4.25, 3.63, and 3.36 kWh/m^2^ for the 1st, 2nd, 3rd, and 4th climate regions, respectively. These values were calculated for the typical days. Thus, annual values were obtained at 1447.5, 1554.4, 1327.95, and 1230 kWh/m^2^ for the 1st, 2nd, 3rd, and 4th regions. These values are far beyond the storage capacity of the PCM-impregnated wood fiber-based insulation material.

### Effect of the PCM on energy saving

**Figure 5:**
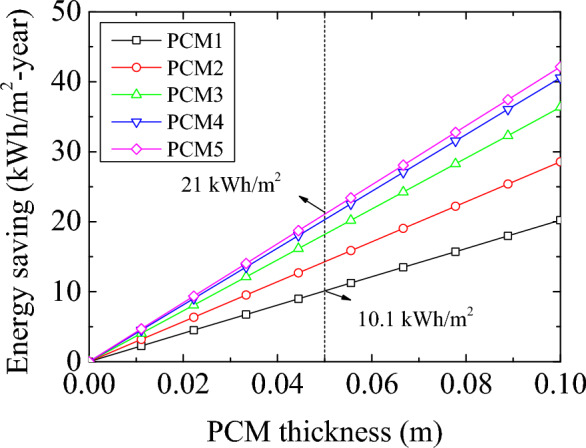
Annual energy saving for PCM1 (25 wt%), PCM2 (35 wt%), PCM3 (45 wt%), PCM4 (50 wt%) and PCM5 (52 wt%).

The solar energy analysis indicates that the energy-storing capability of PCM can be utilized at maximum capacity. The analytical calculation was carried out to evaluate the impact of the novel PCM materials on the building energy performance. PCM-impregnated wood fiber-based insulation material (PCM-INS) was considered for thermal insulation and energy storage. Five different PCM-impregnated insulation materials were applied to the building. The material was varied regarding the thermal storage capacity. The energy storage capacities of PCM-INS panels (0.05 m × 1 m^2^ × 1 m^2^) were calculated to be 60.1 kJ, 84.9 kJ, 108.1 kJ, 120.6 kJ, and 125.3 kJ for PCM1, PCM2, PCM3, PCM4, and PCM5. The sample building is a two-story building. The PCM-INS panels were considered for the walls, ceiling, and floors. The analysis was carried out using the total capacity of the PCM energy storage since solar radiation can provide sufficient energy for the selected location. Four different locations in different climate regions were chosen for evaluation. Figure 5 shows the annual energy saving per unit area of the structure as a function of the PCM-INS thickness.

The thicknesses of PCM-INS varied between 0 and 0.1 m. The amount of energy storage is directly related to the amount of PCM in the wood fiber insulation material. The largest energy-saving capacity belongs to PCM5. It can provide an annual energy saving of 21 kWh/m^2^. It is followed by PCM4 (20.28 kWh/m^2^), PCM3 (18.18 kWh/m^2^), PCM2 (14.28 kWh/m^2^), and PCM1 (10.1 kWh/m^2^), respectively. The great content of the PCM provides outstanding thermal absorption. However, there is another issue that ought to be dealt with. The wood fiber has a thermal conductivity of 0.038 W/m^2^. The eutectic mixture SA-CA PCM has a thermal conductivity of 0.14 W/m^2^. PCM provides an advantage with the thermal energy storage characteristic, while PCM impregnation in the wood fiber reduces the thermal resistance of the insulation with growing thermal conductivity. It has an adverse effect the heat transfer. Therefore, it is required a comprehensive energy performance analysis. Thus, we can decide whether the PCM has an advantage on the building energy performance or is a thrashing.”

The structure's total heat necessity was calculated regarding the thermal gains and losses of the building. The sample building was considered in four climate regions. The heat requirements of buildings in different regions drastically change regarding their environmental and climatic characteristics. Fourier's law of conduction and Newton’s law of cooling calculated the conductive and convective heat losses through the wall, windows, ceiling, doors, and floor. Thermal loss through ventilation was also considered to decide the heat needed. Apart from the thermal storage of PCM, solar energy and interior heat gains were considered in the evaluation. The internal heat gains arise from the heat dissipation of the electrical devices and people in the building.

**Figure 6:**
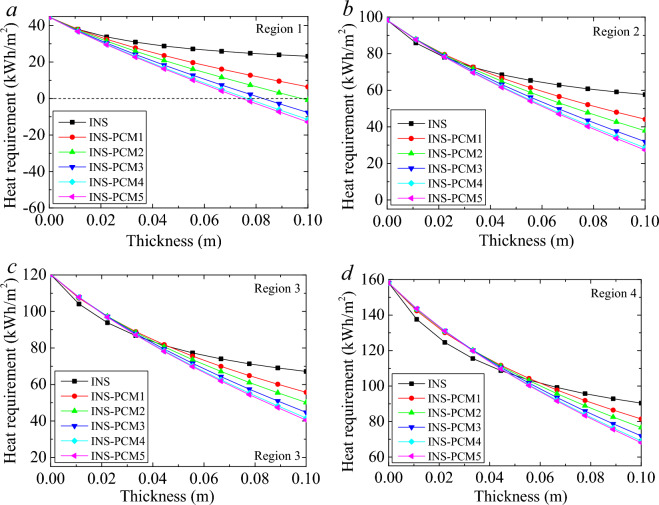
Annual heat requirement for the buildings with only INS and INS-PCMs in the different climate regions.

Figure 6 illustrates the annual heat requirements of six different buildings in four other climate regions. The INS case indicates a building using only insulation without PCM. The heat requirement range in the *y*-axis in the figures was set with the same to see the reduction in the heat requirements. Insulated buildings without PCM show different trends from the other buildings with PCM. The increment in the thickness reduces the heat requirement. However, the reduction in the heat requirement gradually diminishes and becomes even. Therefore, as seen in the figure, the increment of the insulation thickness (without PCM) becomes unnecessary after a certain thickness. In the 1st climate region, the difference in the curve trends becomes apparent with the increment in the thickness.

In Region 2, all cases show similar characteristics until 0.027 m thickness. The difference in the heat requirement between the INS and INS + PCMs gets more significant with an increment in the thickness. In the 3rd and 4th, the advancement of PCM appears after a specific thickness, and it varies according to the Region. The similarity in the amount of the heat requirement continues until 0.025 m in the 3rd Region. Until the thickness of about 0.035 m, INS shows a better performance than that of INS-PCMs. In a clear view, the INS case requires similar heat in the thickness of 0.035 m with PCM3, PCM4, and PCM5 cases, in the thickness of 0.041 m with PCM2, and in the thickness of 0.046 m with INS-PCM1 case. In other words, after these thicknesses, the benefit of the PCM becomes apparent. In the 4th Region, the thermal requirement of all INS-PCM is quite similar until the thickness of about 0.035 m. After that thickness, the difference becomes more apparent. The advantage of PCM appears in a larger thickness in the 4th Region. The heat requirement of the INS case is equal to that of INS-PCM4 and PCM5 cases for 0.048 m. The similarity appears for 0.05 m thickness in the case of INS-PCM3, for 0.054 m thickness in the case of INS-PCM2, and for 0.06 m in the case of INS-PCM1.

**Figure 7:**
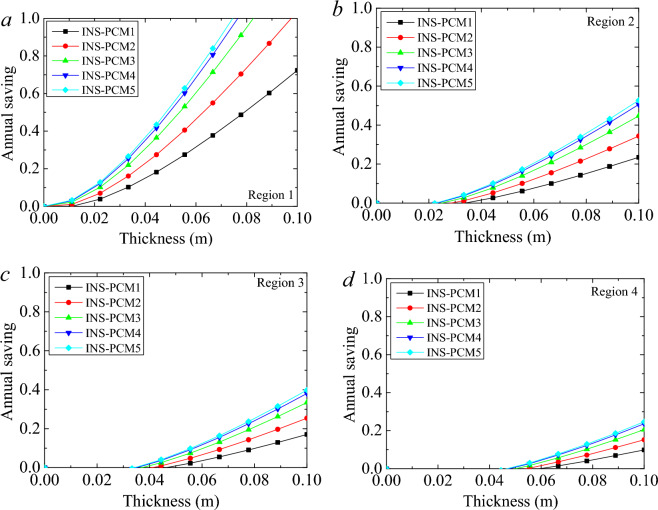
Annual saving rate for all PCMs in the different climate regions.

Similarly, the advantages of PCM appear after these specific thicknesses. As for the energy saving amount of INS case, the reduction of the heat requirement becomes about 21.41 kWh/m^2^ for the thickness variation between 0 and 0.1 m in the 1st climate region. In the case of PCM1, this reduction becomes about 38.2 kWh/m^2^. For the other INS-PCM cases, the maximum possible saving of 44.62 kWh/m^2^ can be attained for a thickness of 0.1 m. In the 2nd Region, the decrease in the thermal requirement for this thickness variation becomes about 40.7 kWh/m^2^ for INS and 71.1 kWh/m^2^ for PCM5. These reductions become 53.1 kWh/m^2^ (for INS) and 79.86 kWh/m^2^ (for PCM5) in the 3rd Region and 67.71 kWh/m^2^ (for INS) and 90.06 kWh/m^2^ (for PCM5) in the 4th Region. The difference in the heat requirement between the INS and INS-PCM5 becomes 23.2, 30.4, 26.72, and 22 kWh/m^2^ for the 1st, 2nd, 3rd, and 4th regions, respectively. These results are exciting. The most considerable reduction in the heat requirement occurs in the fourth Region. However, PCM can provide the most extensive advancement to INS in the 2nd climate region. 3rd, 1st, and 4th regions follow it. This interesting order arises from the fact that the PCM’s energy-storing capability is larger than the energy requirement of the building in the 1st Region. In the 1st Region, it is no need to enlarge the thickness after a certain value. The most attractive case occurs in the first Region due to the zero-energy building potential. As seen in Fig. 7a, the heat requirement drops below zero after a certain thickness. Namely, using the PCM, the heat requirement disappears in the first Region. It is about the low heat needed due to the warm climate characteristic of the Region. It is better to evaluate the annual saving in the Region. The annual saving rate was the ratio of energy saved to the heat required. Figure 7 shows the annual saving rate for all PCMs in the different climate regions. In the 1st Region, the heat requirement can be eliminated for the case of PCM5, PCM4, PCM3, and PCM2. In the case of PCM5, 100% energy saving can be achieved for the INS-PCM thickness of 0.074 m. PCM4 and PCM5 show quite close characteristics due to the relative amount of PCM contents. For PCM4, this thickness becomes 0.076 m. This full saving is achieved for a thickness of 0.083 m and 0.097 m for PCM3 and PCM2, respectively. The energy storage capacity of PCM1 needs to be increased to diminish the heat energy requirement in the 1st Region. On the other hand, it achieved a substantial energy saving of 72.2% for 0.1 m thickness of INS-PCM. The largest energy saving can be achieved for the INS-PCM5 case for all regions.

The effect of PCM appears in the thickness of 0.022 m in the 2nd Region. The saving reaches 52.7% for PCM5 for a thickness of 0.1 m. The PCM1, PCM2, PCM3, PCM4 can provide energy saving rates of 23.5%, 34.3%, 44.7% and 50.5%, respectively. The effect of the PCM appears in the thickness of 0.033 m in the 3rd Region. The saving rates reach 17%, 25.4%, 33.4%, 38% and 39.8% for PCM1, PCM2, PCM3, PCM4 and PCM5, respectively. In the 4th, the saving rate is lower compared to other regions. The effect of PCM becomes apparent for the 0.044 m INS-PCM thickness. The energy saving rates are 9.8%, 15.2%, 20.5%, 23.6%, and 24.7% for 0.1 m thickness in the cases of PCM1, PCM2, PCM3, PCM4, and PCM5, respectively. These comparatively low energy-saving rates are due to harsh winter conditions' high thermal energy requirement. Even these comparatively lower rates are crucial and become significant for cumulative application. Although the lowest thermal resistance is seen in the case of PCM5, it still can provide the largest energy saving. The advantage of energy storage suppresses the adverse effect of the larger thermal conductivity of PCM5 in the 1st regions for all thicknesses and after a certain thickness in the 2nd, 3rd, and 4th regions. These thicknesses are seen in these figures. It may be better to concentrate on one PCM for the evaluation, and it is beyond argument that PCM5 shows the best performance to the other INS-PCMs.

### Effect of the PCM on carbon emission

The building's carbon emissions change depending on the fuel types. It is about the carbon content in the fuel. The complete combustion of the fuels with air was mentioned in the previous section. The amount of carbon emission was calculated to be about 2.7 kg-CO_2_/kg-fuel, 3.2 kg-CO_2_/kg-fuel, 2.64 kg-CO_2_/kg-fuel, and 3.35 kg-CO_2_/kg-fuel for the coal, fuel–oil, natural gas, and LPG, respectively.

The carbon emission of these fuels can be calculated utilizing the combustion reaction. Electricity generation also requires a thermodynamic process. The amount of carbon emission for electricity generation is directly related to the generation methods. Renewable or nuclear energy may diminish carbon emissions without considering the installment or production process of the power plants.

However, natural gas, coal, or fuel–oil-based power plants have an essential share in developing and developed countries. Therefore, assuming electricity as clean energy directly relates to the generation method. In Türkiye, about 37.2% of the electricity generation is obtained by coal-fired power plants. Coal has the largest share. It is followed by NG-fired thermal power plants, with 18.6% in fossil-fuel-based plants. A small percentage belongs to fuel oil with 0.24%. The rest of the generation is hydropower at 29.2%, wind energy at 7.15%, solar energy at 3.16%, geothermal energy at 2.94 and biofuels at 1.49%^63^. Türkiye and Egypt are developing countries with significant investments in renewable energy technology in the last decades^64^.

**Figure 8:**
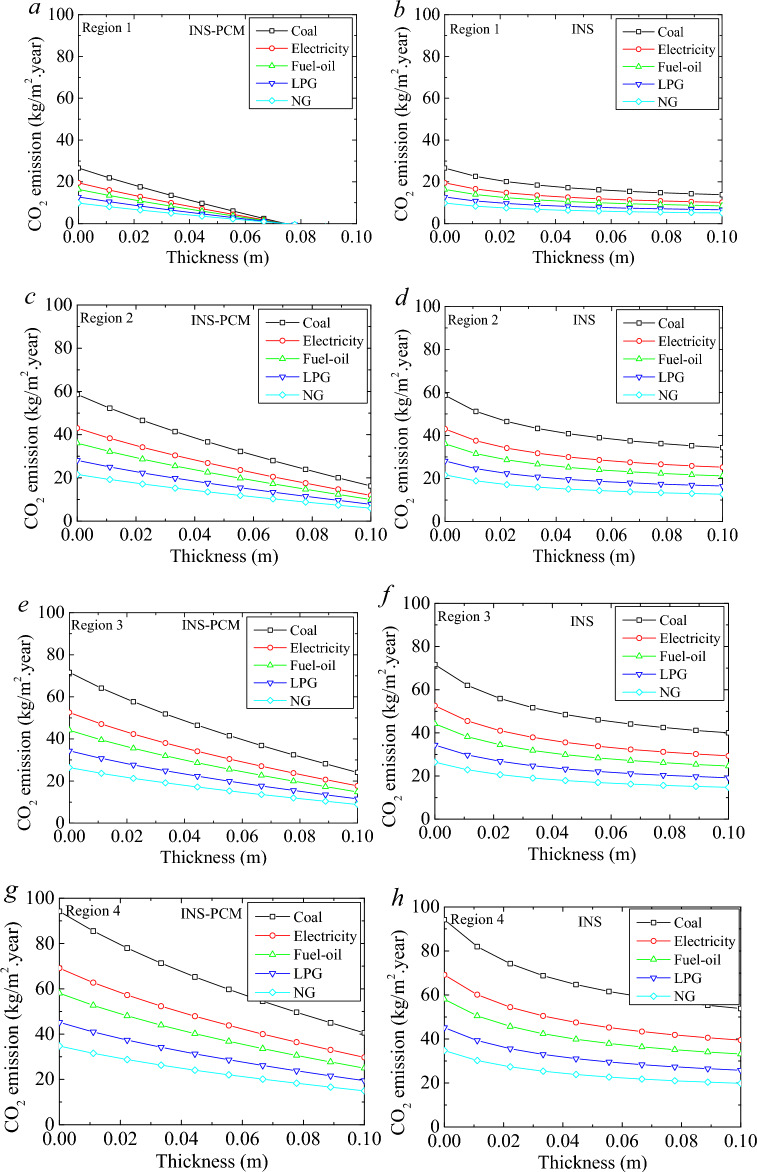
CO_2_ emission for selected fuels in the four climate regions for PCM5.

Therefore, the electricity generation share may change over the years. In this stage, the amount of carbon released for electricity generation can be decided by considering the quantity of the fuels to produce 1 kWh of electricity, the power generation rate for 1 kWh of electricity generation, and the amount of carbon in the fuel used to generate electricity. In the last case, the amount of carbon emission for electricity generation was calculated to be 0.434 kg-CO_2_/kWh. These amounts of carbon emission may be deceiving without considering the low heat values of the fuels. INS and INS + PCM5 cases were considered to investigate the quantity of carbon emissions. Figure 8 illustrates the carbon emission of these two cases for different climate regions and various fuels. As seen in the figure, PCM impregnation can significantly reduce carbon emissions. The effect of the insulation on the carbon emission is mostly clear until a certain insulation thickness. After that, the increment in the insulation thickness becomes ineffective.

In the 1^st^ Region, the carbon emission reduction of the coal-using building becomes 12.75 kg per m^2^ building usage area for the increment of insulation from 0 to 0.1 m without PCM. The PCM-impregnated insulation can provide a 26.58 kg reduction in carbon emission for 0.074 m thickness. It means all carbon emissions from heating purposes can be eliminated for this thickness. For the first Region, considering 0.074 m thickness can give a better comparison. The reduction becomes 11.6 kg for 0.074 m thickness in the case of insulation without PCM. The amount of carbon reduction varies depending on the fuels. In the case of electricity, the reduction becomes 8.5 kg and 19.5 kg-CO_2_/m^2^ for INS and INS-PCM5 cases, respectively. The PCM can provide an 11 kg larger reduction. In the 2nd Region, the reduction of carbon emission enlarges. The decrease in coal becomes 24.25 kg and 42.36 kg-CO_2_/m^2^ for INS and INS-PCM5 cases, respectively. The PCM provides about 18.1 kg-CO_2_/m^2^ more reduction for coal. The advantages of INS-PCM5 compared to the INS become 13.3 kg, 11.1 kg, 8.7 kg, and 6.67 kg-CO_2_/m^2^ for electricity, fuel–oil, LPG, and natural gas cases. In the 3rd Region, the reduction for coal becomes 31.6 kg and 47.5 kg-CO_2_/m^2^ for INS and INS-PCM5 cases. The decrease in carbon emission enlarges for the 3rd Region compared to the 1st and 2nd regions. However, the advancement of PCM in CO_2_ emission reduction gradually decreases for a higher region number. For the case of coal, the improvement becomes 15.9 kg-CO_2_/m^2^ in the 3rd Region. It becomes 13.3 kg, 11.1 kg, 8.7 kg, and 6.67 kg-CO_2_/m^2^ for electricity, fuel–oil, LPG, and natural gas cases. The most considerable reduction in carbon emission and the lowest advancement of PCM compared to the sole insulation case is observed in the 4th Region. The reductions become 40.37 kg and 53.68 kg-CO_2_/m^2^ for INS and INS-PCM5 cases.

The advancement becomes 13.3 kg, 9.77 kg, 8.2 kg, 6.4 kg, and 4.9 kg-CO_2_/m^2^, respectively. The most significant improvement occurs in the case of coal, electricity, fuel oil, LPG, and natural gas, respectively. It is related to the amount of carbon emission. Coal causes the most significant carbon emission. The carbon emission of electricity depends on the source of the generation method. In conclusion, the PCM becomes the most efficacious in the case of the 1st Region, then the 2nd, 3rd, and 4th regions, respectively. In any case, PCM can provide a significant benefit in reducing carbon emissions.

### Effect of the PCM on cost saving

**Figure 9:**
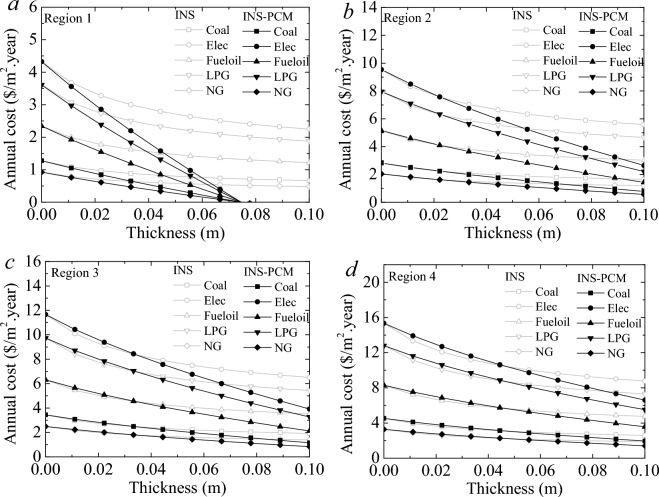
Annual cost of heating for all fuels in four climate regions.

**Figure 10:**
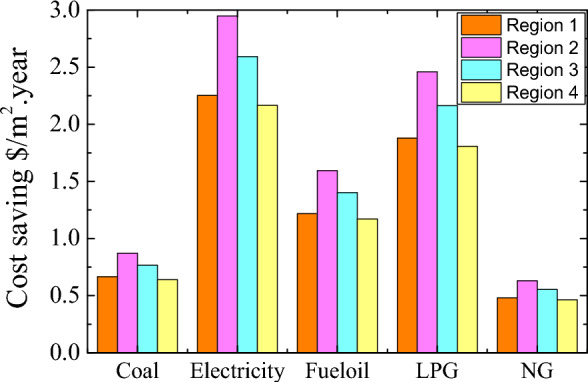
Annual cost saving of INS-PCM5 compared to INS for all fuels in four climate regions.

The effect of PCM on energy and carbon emission decrease was evaluated by adapting PCM-impregnated wood fiber-based insulation material on the building wall. The buildings were considered to use numerous fuels in four climate regions. Energy and carbon emission saving are vital issues. However, an investment can be reasonable when it is cost-effective to utilize. Individual utilizers are one of the most important shareholders of the energy-saving strategy for sustainability. The cost-saving rate changes regarding the price of the energy source. This study considered coal, electricity, fuel oil, LPG, and natural gas, and their current prices were used to decide the cost saving. The price averages of the fuels were 0.25 $/Nm^3^, 0.13 $/kg, 0.096 $/kWh, 0.46 $/kg, 0.95 $/kg for natural gas, coal, electricity, fuel oil, and LPG, respectively, in Türkiye and Egypt. Figure 9 shows various regions' annual heating costs per unit area. INS and INS-PCM5 using buildings were considered for the evaluation. The figure illustrates the annual price of the heating without considering the PCM price. The figures show that the PCM can provide the most apparent advantage in the 1st Region. Insulation is the most effective and significant part of the energy-saving goal. However, increments in the thickness never eliminate the energy loss but reduce heat transfer. The annual cost becomes straight after a certain increment of the insulation thickness. Namely, its effect on cost-saving performance becomes insignificant and unnecessary. However, PCM can provide a direct advantage due to its energy storage feature. A larger thickness of the INS-PCM means larger energy storage and lower energy need. The largest heating price belongs to electricity. LPG follows it. It is about the amount of energy needed and the price. Therefore, the major cost saving arises in the electricity case. The annual heating cost is eliminated for the INS-PCM5 thickness of 0.074 m. For this thickness, the heating cost for coal decreases by 0.56 $ and 1.28 $/m^2^.year for INS and INS-PCM5 cases. These reductions become about 1.89 $ and 4.32 $/m^2^.year for electricity. INS-PCM5 can provide about 2.28 times larger energy saving for all fuels. In the 1st Region, INS-PCM5 provides 0.72 $ (for coal), 2.43 $ (for electricity), 1.3 $ (for fuel–oil), 2.03 $ (for LPG), and 0.52 $/m^2^.year saving (for natural gas) compared to INS (Fig. 10). In the 2nd, 3rd, and 4th regions, the advantages appear after a certain thickness; these thicknesses are 0.022 m, 0.033 m, and 0.044 m, respectively. INS-PCM5 can provide about 1.74-, 1.5-, and 1.33 times larger energy saving than INS in the 2nd, 3rd, and 4th regions for all fuels. In the 2nd Region, the savings become 0.87 $ (for coal), 2.94 $ (for electricity), 1.59 $ (for fuel–oil), 2.46 $ (for LPG), and 0.63 $/m^2^ per year (for natural gas) (Fig. 10). These become 0.76 $, 2.59 $, 1.4 $, 2.16 $, and 0.55 $/m^2^. year in the 3rd region and 0.64 $, 2.16 $, 1.71 $, 1.8 $, and 0.46 $/m^2^. year in the 4th region, respectively. In the 2nd Region, the savings become larger than in the 1st Region. On the other hand, the amount of savings is reduced in the 3rd and 4th Regions. Although a larger energy saving was achieved in the 2nd Region, using PCM in the first Region is preferable due to zero energy building potential and less INS-PCM thickness. The amount of cost savings enlarge for a building with larger usage areas.

**Figure 11:**
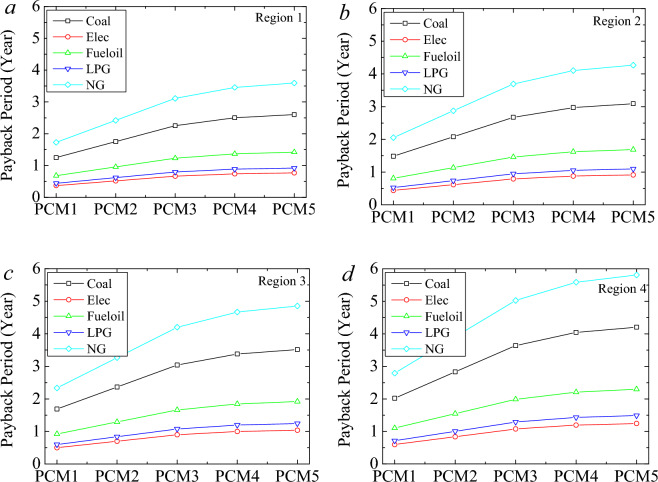
Payback period of the phase change materials (0.1 m) for various fuels.

Stearic and capric acid prices could be high for a laboratory-scale application. However, the cost of the PCM can be significantly reduced for bulk purchase of the acids. Thus, the investment becomes highly conceivable. For the payback period evaluation, the bulk purchase of the acids was considered. Figure 11 shows the payback period of the phase change materials (0.1 m) for various fuels. The increment in the thickness of INS-PCM provides a more significant amount of PCM impregnation, and this larger PCM can provide more energy storage. INS-PCM5 requires a more extended payback period than the other PCM cases.

On the other hand, it also can provide a larger annual energy and cost savings. The shortest payback period is observed in the building using electric heating. The payback period varies from 0.37 to 1.24 years PCM1 case in the 1st Region and PCM5 case in the 4th Region, respectively, for the electricity case. After that, the building using LPG for heating can provide the second shortest payback period. It varies between 0.44 and 1.48 years for LPG for PCM1-Region1 and PCM5 Region 4. The most extended payback period is observed for the case of natural gas. It varies between 1.72 and 5.8 years for the same issues.

The coal case follows it and varies between 1.25 and 4.2 years. The longest and shortest payback period becomes 0.37–5.81 years regarding the fuel and Region.

## Conclusion

Wood fiber is a significant potential supportive component for creating a new composite PCM due to its beneficial qualities, including high sorption competency, low density, enviro -friendliness, economic effectiveness, and chemical inertness. This study used a stearic-capric acid/wood fiber (SA-CA) eutectic mixture for thermal energy storage, which experiences a phase change within the building's thermally pleasant temperature range. The study examines the PCM impact on building energy performance for net-zero energy building potential. A heat transfer investigation was conducted to state the building energy performance by considering the heat losses through the building components and ventilation, the heat gains through solar and inner gains, and the thermal energy storage of PCM. Five different PCM-impregnated wood fiber-based insulation materials were applied to the sample buildings in four different climate regions. The advantages of INS-PCMs were investigated in typical insulated buildings. The energy, fuel, cost, and carbon emission saving capacities were decided regarding the fuels. The following results summarize the study.

1. The largest energy-saving capacity belongs to PCM5. It can provide an annual energy saving of 21 kWh/m^2^. PCM provides an advantage with the thermal energy storage characteristic, while PCM impregnation in the wood fiber reduces the thermal resistance of the insulation by increasing thermal conductivity. Therefore, it is vital to consider the energy-saving potential of PCM along with the increment in the heat transfer rate.
2. The energy analysis indicated that the PCM-impregnated insulation material provides a significant advantage compared to the conventional insulation case in the first Region. The difference in the heat requirement between the INS and INS + PCMs gets more critical with an increment in the thickness. In the 3rd, the conventional insulation (INS) performs better than the INS-PCMs integrated building until the thickness of about 0.035 m. In the 4th region, this thickness becomes 0.048 m. The advantages of PCM appear after specific thicknesses.
3. The difference in the heat requirement between the INS and INS-PCM5 becomes 23.2 kWh/m^2^, 30.4 kWh/m^2^, 26.72 kWh/m^2^, and 22 kWh/m^2^ for the 1st, 2nd, 3rd, and 4th regions, respectively. This exciting order arises from the fact that the PCM’s energy storage capacity is larger than the energy requirement of the building in the 1st Region. In the 1st Region, it is no need to enlarge the thickness after a specific value. The most attractive case occurs in the first Region due to the zero-energy building potential.
4. The energy saving reaches 52.7% for PCM5 for a thickness of 0.1 m. The PCM1, PCM2, PCM3, PCM4 can provide energy saving rates of 23.5%, 34.3%, 44.7% and 50.5%, respectively.
5. The carbon emission analysis showed that the advancement of PCM in CO_2_ emission reduction gradually decreased for a higher region number. The largest reduction in carbon emission occurs in the case of coal, electricity, fuel oil, LPG, and natural gas, respectively.
6. The PCM-impregnated insulation can provide a 26.58 kg reduction in carbon emission for 0.074 m thickness. In the 2nd Region, the PCM offers about 18.1 kg-CO_2_/m^2^ more reduction for coal.
7. The most significant reduction in carbon emission and the lowest advancement of PCM compared to the sole insulation case is observed in the 4th Region. In conclusion, the PCM becomes the most efficacious in the case of the 1st Region, then the 2nd, 3rd, and 4th regions, respectively. In any case, PCM can provide a significant advantage in reducing carbon emissions.
8. The cost analysis showed that, in the 1st Region, INS-PCM5 provides 0.72 $, 2.43 $, 1.3 $, 2.03 $, and 0.52 $/m^2^ year saving compared to INS for coal, electricity, fuel oil, LPG, and natural gas, respectively. In the 2nd, 3rd, and 4th regions, the advantages appear after a certain thickness; these thicknesses are 0.022 m, 0.033 m, and 0.044 m, respectively. INS-PCM5 can provide about 1.74-, 1.5-, and 1.33 times larger energy saving than INS in the 2nd, 3rd, and 4th regions for all fuels.
9. The shortest payback period is observed in the building using electric heating. INS-PCM5 requires a more extended payback period compared to the other PCM cases.

The most extended payback period is observed for the case of natural gas. It varies between 1.72 and 5.8 years for the same issues. The coal case follows it and varies between 1.25 and 4.2 years. The payback period varies between 0.37 and 5.81 years regarding the fuel and Region.

## Data Availability

The datasets used and/or analyzed during the current study are available from the corresponding author upon reasonable request.
